# Subtypes of Melanomas Associated with Different Degrees of Actinic Elastosis in Conventional Histology, Irrespective of Age and Body Site, Suggesting Chronic Ultraviolet Light Exposure as Driver for Lentigo Maligna Melanoma and Nodular Melanoma

**DOI:** 10.3390/cancers16010001

**Published:** 2023-12-19

**Authors:** Konstantin Drexler, Veronika Zenderowski, Laura Schreieder, Kevin Koschitzki, Sigrid Karrer, Mark Berneburg, Sebastian Haferkamp, Dennis Niebel

**Affiliations:** Department of Dermatology, University Medical Center Regensburg, 93053 Regensburg, Germanylaura.schreieder@ukr.de (L.S.); kevin-thomas.koschitzki@klinik.uni-regensburg.de (K.K.); sigrid.karrer@ukr.de (S.K.); mark.berneburg@ukr.de (M.B.); sebastian.haferkamp@ukr.de (S.H.); dennis.niebel@ukr.de (D.N.)

**Keywords:** actinic elastosis, melanoma, nevogenesis, carcinogenesis, histopathology

## Abstract

**Simple Summary:**

Increased sun exposure and sunburns lead to higher numbers of moles as well as melanomas and non-melanoma skin cancers. Scientists are unsure whether there is a difference between being in the sun very often (chronic sun damage) and being in the sun for too long at certain times (intermittent exposure) in terms of the individual melanoma risk. In this study, we used light microscopy to look at typical connective tissue changes in the skin that occur with long-term sun exposure. We analyzed whether these changes are correlated with different subtypes of melanomas and whether they are associated with sun-exposed body sites (chronic exposure) and shaded body sites (intermittent exposure). Our results show that tissue changes near moles and melanomas as well as subtypes of melanomas vary, regardless of patient age and tumor site. This finding is important because it sheds light on the biological effects of sunlight on pigment cells, which are the source of moles and melanomas. Moreover, it emphasizes the need to more clearly differentiate among the subtypes of melanomas.

**Abstract:**

(1) Background: Ultraviolet (UV) radiation and sunburns are associated with an increased incidence of acquired nevi and melanomas. However, the data are controversial as to whether chronic UV exposure or high intermittent UV exposure is the major carcinogenic factor in melanocytic tumors. In this study, we compared the degree of actinic elastosis (AE) as a surrogate for lifetime UV exposure in nevi and different clinical melanoma subtypes (i.e., superficial spreading melanoma (SSM), nodular malignant melanoma (NMM), acral lentiginous melanoma (ALM), and lentigo maligna melanoma (LMM)) with respect to clinical variables (age, sex, and body site). (2) Methods: We defined a semi-quantitative score for the degree of AE ranging from 0 = none to 3 = total loss of elastic fibers (basophilic degeneration) and multiplied it by the perilesional vertical extent (depth), measured histometrically (tumor-associated elastosis grade (TEG)). We matched the TEG of *n* = 595 melanocytic lesions from 559 patients with their clinical variables. (3) Results: The TEG was correlated with age and UV-exposed body sites. Furthermore, the TEG was significantly higher in LMM than in all other types of melanomas and the TEG in NMM was higher than in SSM, irrespective of patient age and tumor site. (4) Conclusions: High cumulative UV exposure is more strongly associated with LMM and NMM than with other melanoma subtypes.

## 1. Introduction

It is widely accepted that radiation and ultraviolet (UV) light exert numerous biological effects on skin cells, including keratinocytes, melanocytes, and immune cells. Depending on the amount of melanin in the skin, people have a limited endogenous protective capacity against UV radiation. Excessive UV exposure may cause dermatitis solaris (sunburn), with fair-skinned individuals being at the highest risk. Daily exposure to high levels of UV radiation without the development of dermatosis solaris may induce irreversible changes, such as an increased melanocyte density and activity [1]. Both repeated sunburns and high cumulative UV exposure may be risk factors for the development of skin cancer, including melanomas and non-melanoma skin cancer (NMSC) [2,3]. However, there are subtypes of melanomas that occur independently of UV exposure, such as acral lentiginous melanoma (ALM) and mucocutaneous melanoma. Therefore, a more detailed look at benign melanocytic lesions and the subtypes of melanomas is required. The slightest hyperplasia of melanocytes in the skin are ephelides, which are largely genetically determined, but also UV-inducible [4]. Lentigines, on the other hand, are signs of photodamage and occur in chronically sun-exposed areas, such as the dorsal arms and hands [5]. Lentigines may be caused by the permanent stimulation of melanocytes, which also explains the uneven pigmentation (poikiloderma) of the surrounding skin [6]. Melanocytic nevi may be congenital, but are more commonly acquired, and are all benign tumors. The current evidence indicates that either chronic UV exposure or high intermittent UV exposure, including sunburns, may lead to the development of nevi (distinct pathways) [7,8]. However, nevi decrease from the age of 30. One study suggested that chronic solar damage (CSD) is associated with a lower number of nevi [9,10], but a more recent study failed to reproduce these findings [11]. For cutaneous melanomas, the malignant counterpart to acquired nevi, the data on the effects of chronic and intermittent UV exposure are even more puzzling. There is strong evidence that high intermittent UV exposure, especially during childhood and adolescence, is a major risk factor for cutaneous melanomas [12]. However, a large registry-based study showed that cutaneous melanomas are more commonly found in the head and neck area than in other anatomical regions, which may point to CSD as a pathophysiological trigger [13]. Surprisingly, in another large cohort study of 966 individuals, high chronic UV exposure was associated with several types of NMSC, but not with melanomas [11]. A recent population-based cross-sectional study showed that the site of the melanoma is an important prognostic factor, with lower-extremity melanomas most likely to have loco-regional nodal spread and head and neck melanomas most likely to be stage IV at the time of the diagnosis [14]. These conflicting findings led to the theory that there are different UV-driven patterns of melanoma development. Whiteman et al. discussed an individual tendency to develop melanomas of the trunk with multiple nevi, associated with high intermittent UV exposure, versus head and neck melanomas with multiple lentigines in the course of CSD [15]. Brozyna et al. proposed an additional mode in 2007 to encapsulate three distinct pathways of melanoma genesis [16]:

1. Intermittent sun exposure and the tendency to develop multiple melanocytic nevi;
2. Multiple sunburns during childhood and general sun sensitivity with freckling (ephelides);
3. Chronic sun exposure and the development of multiple lentigines.

However, to date, few studies have clearly separated the clinical subtypes of cutaneous melanomas, the associated pathways, and the body sites to stratify the effects of different types of UV exposure. Traditionally, melanomas have been classified as superficial spreading melanoma (SSM), nodular melanoma (NMM), acral lentiginous melanoma (ALM), and lentigo maligna melanoma (LMM). It is tempting to assign SSM and NMM to pathways 1 and 2 and LMM to pathway 3, with ALM being largely UV-independent. However, this classification has not yet been studied in detail. Since the Human Genome Project, a genomic classification of cutaneous melanomas has also been in use (mutant BRAF, mutant NRAS, mutant NF1, and triple wildtype) [17], which is of great importance for therapeutic decisions in metastatic settings. In this study, we will mainly focus on the clinical subtypes of melanomas to allow comparisons with historical data. Literature reports focusing on the different clinical subtypes of melanomas and different aspects of UV exposure are sparse. An Italian study found that the total number of nevi and a higher level of education and sunburns in childhood are more strongly related with SSM than with other melanoma subtypes [18]. Consistent with this, an Australian case-control study found a higher risk of SSM with sunburns and the total number of nevi, indicating high intermittent UV exposure as a major risk factor, whereas LMM was associated with lentigines and a history of NMSC, indicating CSD as the main risk factor [19]. A French group also differentiated LMM from other types of melanomas with respect to these risk factors [20].

Conventional histology is a viable method for estimating CSD because prolonged exposure to UV light is reflected by actinic elastosis (AE) in the specimen [21]. The degree and depth of AE is correlated with patient age and sun-exposed body sites, as shown previously [22]. Immunohistological markers, on the other hand, may be suitable for detecting the acute effects of solar skin injury [23]; at present, however, there is no immunohistochemical panel superior to standard hematoxylin and eosin (HE) staining for quantifying AE. In a previous study, we were able to show significant differences in the peritumoral extent of AE between cutaneous squamous cell carcinoma (cSCC) and basal cell carcinoma [22]. When examining the degree of AE (semi-quantitative four-point scale) and the presence of nevi remnants in the vicinity of 500 cutaneous melanomas, Kvaskoff et al. found significant differences between melanomas of the trunk and melanomas of the head and neck. However, they excluded LMM from their analysis [24]. Lee et al. conducted a smaller study of 141 melanomas (95 SSM and 46 LMM) to find a higher degree of AE in melanomas of the head and neck than in melanomas of the back [25]. 

In this study, we extended our previously established method to melanocytic tumors to investigate the differences between the clinical subtypes of melanomas in more detail. Our main hypothesis was that LMM tends to have more peritumoral AE and is more likely to occur on the head and neck (CSD pathway), whereas SSM tends to have less peritumoral AE and is more likely to occur on non-UV-exposed body sites (intermittent UV exposure pathway). In addition, we investigated the differences in CSD between nevi and melanomas and other subtypes of melanomas.

## 2. Materials and Methods

### 2.1. Patient Characteristics and Inclusion Criteria

For the analysis of AE, we selected the most recent (*n* = 700) cases of melanocytic tumors at the University Medical Center of Regensburg from 2014 to 2022, with a proportion of *n* = 500 melanomas, including SSM, NMM, and ALM (100 in each stage of disease; IA-IIC); *n* = 100 LMM (any stage); and *n* = 100 nevi. After screening, 105 cases had to be excluded (20 SSM, 41 NMM, 3 ALM, 9 LMM, 24 unspecified melanomas, and 8 nevi) due to the poor quality of the histopathological specimen or the lack of peritumoral tissue to allow adequate AE grading. Clinical data, including the patient’s age at the time of the diagnosis, their sex, the body site, the Breslow thickness, the tumor stage, ulceration, consecutive systemic therapies, and the BRAF mutation status, were extracted using the i.s.h.med software (Cerner Corporation, North Kansas City, MO, USA, run via SAP 6.0 software, SAP SE, Walldorf, Germany), which is used as hospital management software at our institution. The face, head, neck, hands, and dorsal forearms were defined as “UV-exposed” body sites. There was insufficient clinical information on the total number of nevi, solar freckles, and the presence of multiple lentigines in a given patient, and on the skin phototype and the presence or absence of iatrogenic immunosuppression; hence, these variables could not be included in the analysis.

### 2.2. Histopathological Assessment

The sections were processed according to a standard protocol and stained with HE. The histological examination was performed independently by two experienced dermatopathologists (K.D. and D.N.). The slides were sorted chronologically (date of excision) rather than by tumor type to ensure that the measurement was as unbiased as possible. To assess the depth of AE, the widest identifiable elastotic fiber or area of basophilic degeneration in the vicinity of the tumor and in the absence of the tumor stroma was measured orthogonally from the stratum granulosum using an ocular scale. To assess the degree of AE, a semi-quantitative score was established as follows: 0 = absent, 1 = low: less elastotic material than regular fibers (collagenous and elastic), 2 = moderate: more elastotic fibers than regular fibers, and 3 = strong: complete or almost complete loss of normal fibers or homogeneous basophilic zone. If these scores were consistently within a range of 1 point for the semi-quantitative score and a range of 20% for the AE depth measurement, the mean was calculated and used for subsequent analyses. The agreement between the two raters was moderate for the depth and degree of AE, indicating that the mean of the two raters was a good choice to obtain reliable values. Discrepant results were resolved using a discussion microscope. The depth was multiplied by the semi-quantitative score, which we defined as the tumor elastosis grade (TEG), as described previously [22]. Curettage material, punch biopsies, and specimens without surrounding normal tissue were excluded from further analyses. In the end, a total of *n* = 595 specimens from *n* = 559 patients were included in the statistical analysis.

### 2.3. Statistical Analysis

Statistical tests were performed using IBM SPSS, version 25 (Armonk, NY, USA). The degree of AE and the TEG between different melanocytic tumors was compared using Student’s *t*-test. Multiple regression and an analysis of variance (ANOVA) were used to evaluate the impact of clinical variables (age at the time of the diagnosis and UV-exposed sites). The results were considered statistically significant at *p* ≤ 0.05. Subgroups were compared (LMM vs. all other melanoma subtypes; NMM vs. SSM). These subsequent analyses were chosen because LMM is known to show features of CSD. NMM and SSM are the most common clinical melanoma subtypes, so we wanted to analyze them separately.

### 2.4. Microscope and Digital Photography

Both dermatopathologists used an Olympus BX43 microscope (Olympus, Shinjuku, Japan) for their analyses. All photomicrographs were taken after scanning the slides using a PreciPoint M8 microscope and scanner with the ViewPointLight software, version 1.0.0.9628, for imaging (PreciPoint GmbH, Freising, Germany); we refrained from digital enhancement. The figures were generated using MS PowerPoint Professional Plus 2016, version 16.0.4266.1001 (Microsoft Corp., Redmond, WA, USA).

## 3. Results

### 3.1. Clinical Characteristics and Histopathological Analysis: Nevi vs. Melanomas

To assess the degree of AE in a meaningful and unbiased cohort of patients, we selected the 700 most recent specimens with a diagnosis of a melanocytic tumor from 2014 to 2022. We also balanced the proportion as follows: 100 melanomas (LMM, NMM, and ALM) per stage of disease (IA-IIC); 100 LMM; and 100 acquired benign nevi (including junctional, compound, and dermal nevi). After the exclusion of cases with poor-quality or missing clinical data, 595 melanocytic lesions from 559 patients were included in the analysis. The clinical variables of the study cohort are shown in Table 1.

**Figure 1:**
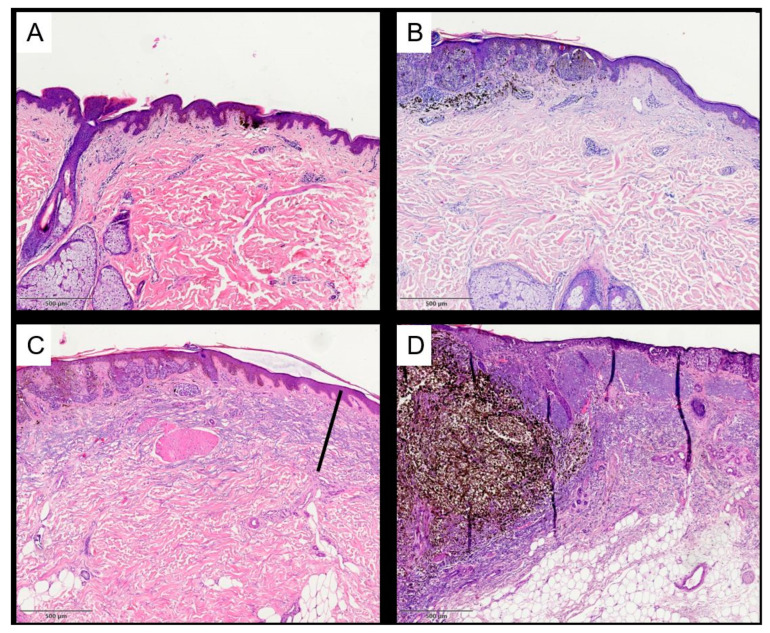
Example of histopathological assessment (tumor-associated elastosis grade—TEG) and measurement of AE depth. The depth of elastotic material was measured in the vicinity of the tumor in the absence of the tumoral stroma. The semi-quantitative score was defined as follows: 0 = absent, 1 = low: less elastotic material than regular fibers (collagenous and elastic), 2 = moderate: more elastotic fibers than regular fibers, and 3 = strong: complete or almost complete loss of normal fibers or homogeneous basophilic zone. Scale bars indicate 500 µm. (**A**) Example of score 0; (**B**) example of score 1; (**C**) example of score 2 and exemplified measurement of the depth of AE; and (**D**) example of score 3.

**Figure 2:**
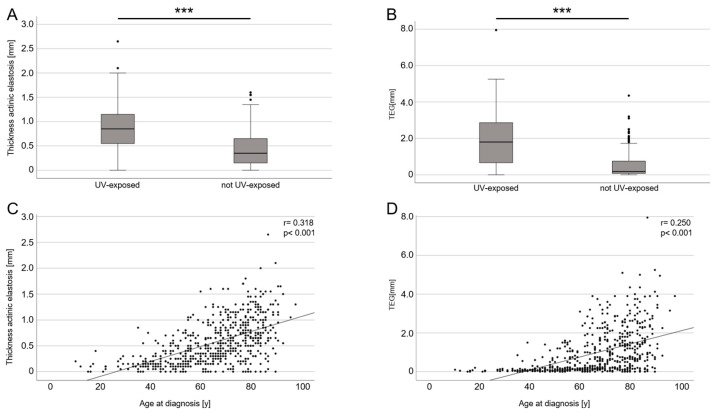
Mean thickness (depth) of AE and TEG correlated with UV-exposed body sites and age at time of diagnosis in all groups combined (nevi and melanomas). (**A**) Boxplot diagram of the mean depth of AE, showing differences between UV-exposed and non-UV-exposed body sites (*p* < 0.001; *** stands for *p* < 0.001). (**B**) Boxplot diagram of TEG (depth × semi-quantitative score), showing differences between UV-exposed and non-UV-exposed body sites (*p* < 0.001; *** stands for *p* < 0.001). (**C**) Point scatter plot, showing a significant correlation (*p* < 0.001) between mean depth of AE and age at time of tumor diagnosis (defined as time of excision). (**D**) Point scatter plot, showing a significant correlation (*p* < 0.001) between TEG and age at time of tumor diagnosis (defined as time of excision).

There was a female predominance in the nevus group (67.4%) and a male predominance in the melanoma group (58.8%). The age at the time of the diagnosis differed by around 23 years between patients with nevi and melanomas. This finding partly explains the differences in the depth of AE and the TEG between nevi and melanomas. UV-exposed body sites were more often affected in the melanoma group (31.6%) than in the nevus group (17.4%), but both groups showed a higher rate of lesions in non-UV-exposed body sites. For all lesions, the depth and grade of AE were analyzed separately by two raters (examples of each grade are shown in Figure 1). The mean depth of AE and the TEG were significantly higher in melanomas than in nevi (*p* ≤ 0.001/*p* ≤ 0.001). However, when a multiple linear regression analysis was used to adjust for clinical variables in the group composition, the difference in AE and the TEG between nevi and melanomas was no longer statistically significant (*p* = 0.765/*p* = 0.508). Consistent with our previous data, UV-exposed body sites (*p* < 0.001) and the age at the time of the diagnosis (*p* < 0.001) were the main variables for the depth of AE and the TEG (Figure 2). This finding applied equally to both men and women.

### 3.2. Clinical Characteristics and Histopathological Analysis: Clinical Melanoma Subtypes

Because the main objective of this study was to compare the depth of AE and the TEG between clinical subtypes of melanomas, we performed subsequent comparisons. A total of 91 LMM and 412 melanomas of other subtypes were analyzed. LMM significantly differed from other clinical melanoma subtypes, as shown in Table 2.

**Figure 3:**
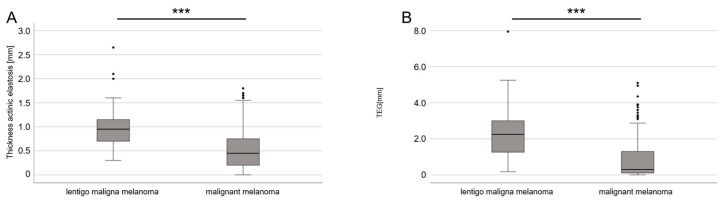
Mean thickness (depth) of AE and TEG, comparing LMM with other melanoma subtypes. (**A**) Boxplot diagram of the mean depth of AE, showing differences between LMM and other melanoma subtypes (*p* < 0.001; *** stands for *p* < 0.001). (**B**) Boxplot diagram of TEG (depth × semi-quantitative score), showing differences between LMM and other melanoma subtypes (*p* < 0.001; *** stands for *p* < 0.001). Abbreviations: AE—actinic elastosis; TEG—tumor elastosis grade.

The majority of patients with melanomas were male (51.6% in LMM versus 60.4% in other melanoma subtypes). Patients with LMM were, on average, 8.9 years older. A total of 84.6% of the patients had tumors located on UV-exposed body sites, compared to only 19.9% of other melanoma subtypes on UV-exposed body sites. The mean thickness of AE and the TEG were significantly higher in LMM than in other melanoma subtypes (*p* ≤ 0.001/*p* ≤ 0.001; Figure 3). These differences in the depth of AE (*p* = 0.008) and the TEG (*p* < 0.001) remained statistically significant when age and UV-exposed body sites were included in the calculation using a multiple regression analysis. A significant effect on AE depth was found for UV-exposed body sites (regression coefficient: 0.285), a diagnosis of LMM (regression coefficient: 0.123), and the age at the time of the diagnosis (regression coefficient: 0.012). In addition, the TEG was correlated with all three parameters (*p* < 0.001). UV-exposed body sites showed an effect (regression coefficient: 1.031), as did a diagnosis of LMM (regression coefficient: 0.521) and the age at the time of the diagnosis (regression coefficient: 0.027; Table 3). When looking at the tumors with a low TEG (≤0.5), we found an equal number of SSM and NMM, followed by nevi and only a few cases of LMM. 

**Figure 4:**
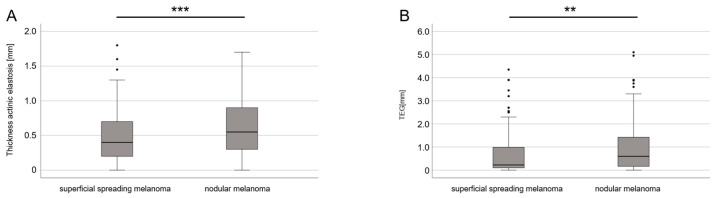
Comparison of thickness (depth) of AE and TEG between SSM and NMM. (**A**) Boxplot diagram of depth of AE in SSM and NMM (*p* < 0.001). (**B**) Boxplot diagram of TEG in SSM and NMM (*p* = 0.006). ** stands for *p* < 0.005, *** stands for *p* < 0.001. Abbreviations: AE—actinic elastosis; TEG—tumor elastosis grade; SSM—superficial spreading melanoma; NMM—nodular malignant melanoma.

As SSM and NMM are regarded as the most common clinical subtypes of melanomas, the thickness of AE and TEG were compared in more detail. The mean thickness of both AE (0.4734 mm vs. 0.6357 mm; *p* < 0.001) and TEG (0.6838 mm vs. 0.9631 mm; *p* = 0.006) was significantly lower in 167 SSM than in 199 NMM (Figure 4). When considering UV-exposed body sites and the age at the time of the diagnosis using a multiple regression analysis, these differences remained statistically significant for the thickness of AE (*p* = 0.007), but not for the TEG (*p* = 0.246; Table 4).

No significant results or trends were found when comparing the tumor stage, the presence or absence of ulcerations, the BRAF mutation status, or the clinical course with the depth of AE or the TEG. ALM showed a lower depth of AE and a lower TEG, but since only 27 ALM (5.4%) were included in the analysis, these results were not statistically significant.

## 4. Discussion

Our results show the importance of distinguishing between chronic and intermittent sun exposure in the development of melanocytic lesions. Also, melanomas must be differentiated according to their clinical subtypes, which have different histopathologic features and a different clinical course. The most important finding of our study is the difference in the depth of AE and the TEG between SSM and NMM, with the mean thickness of AE and the TEG being significantly lower in SSM than in NMM. This finding is surprising for two reasons: (1) it has not been previously reported in the literature, and (2) it did not correlate with the tumor stage, although NMM is usually diagnosed at higher stages than SSM and has a worse prognosis [26].

The second important finding of our study was that we were able to confirm an association between CSD and LMM as opposed to other clinical subtypes of melanomas. This finding is consistent with the theory that LMM occurs with multiple lentigines in the course of lifelong UV exposure, whereas other melanomas tend to be associated with a painful sunburn on non-UV-exposed body sites [16]. 

The third important finding of our study was that both nevi and melanomas are related to UV damage, which is consistent with the literature cited in the introduction [7,9,16]. The fact that the nevus and melanoma groups differed in patient age and sex may indicate a tendency to excise more suspicious lesions in younger patients and in women in Regensburg, while older patients undergo excision only when the lesion is highly suspicious for melanomas. This issue needs further investigation.

Because our newly established TEG score was directly correlated with the age at the time of the diagnosis and UV-exposed body sites, we assume that it is a useful tool as a surrogate for CSD. This assumption is consistent with previous findings by other authors [27]. To put our current results into perspective, we compared similar approaches by other authors. One group used a Verhoeff–Van Giesson stain to quantify elastotic material in the vicinity of skin tumors, but did not examine melanomas [28]. In an Australian study, the author used an HE-based approach similar to ours to differentiate melanomas related to CSD from nevus-associated melanomas in 1200 patients, but only used a dichotomous score (elastosis absent or present) [29]. The author found a strong correlation of AE with age. Similar to our results, he did not find any differences in melanomas with a high degree of AE with respect to the tumor thickness, the mitotic rate, ulceration, or the overall survival time compared to melanomas without AE. The association of AE with age was also found in another study that examined AE in more than 2000 melanomas using a 4-level semi-quantitative score in HE-stained specimens [30]. These authors paid specific attention to the body site and ambient UV exposure, but not to the clinical subtypes of melanomas. Interestingly, in a German case-control study, AE was more strongly associated with melanomas than with basal cell carcinoma, again raising the question of missing information on melanoma subtypes [31]. When looking at studies dealing with this topic, it is noteworthy that AE may bias the interpretation of melanocytic lesions, as it is a diagnostic criterion for dysplastic tumors [32]. Robust connective tissue changes may complicate the diagnostic accuracy in melanocytic tumors [33]. The presence of AE may lead dermatopathologists to diagnose melanomas more easily, which needs to be taken into account when interpreting our data. 

With regard to the genomic classification of melanomas, there was no propensity for BRAF, NRAS, or NF1 mutations in any of the clinical subgroups in this study, although some authors suggest that BRAF mutations are associated with the nevogenic pathway of melanoma genesis and that NF1, ROS1, GNA11, and RAC1 mutations are associated with CSD-associated melanomas [34]. Our data were not sufficient to validate these findings, as the mutational status was available for only 105 melanomas (BRAF mutant: 44, BRAF wildtype: 61).

Our study has several limitations. First, because of the monocentric study design, there may be a bias in the diagnostic trends regarding melanocytic lesions. However, the diagnoses were made by experienced dermatopathologists, most of whom were present at our institute throughout the study. Second, we could not include other clinical data such as the total number of nevi, occupational or recreational sun exposure habits, medication, or the co-occurrence of NMSC, because these data were not sufficiently and consistently available from the patients’ medical records. These factors could be addressed by extending this study to a prospective setting in the future to validate our findings. Third, our technique of assessing CSD by estimating AE in conventional histology lacks the control of a second independent technique such as immunohistochemistry, which has been reported to be useful in the past [35]. Other tools to assess skin aging may be useful to validate AE as a surrogate for CSD, such as the SCINEXA [36,37], which could be addressed in upcoming studies. Nevertheless, as mentioned before, the strength of our study is that we investigated in detail the differences between the clinical subtypes of melanomas, which adds significant information to allow a detailed interpretation of the available data in the literature.

## 5. Conclusions

In summary, our findings suggest that LMM is a distinct subset of melanoma associated with CSD and that NMM is more strongly associated with chronic UV exposure than SSM. CSD was independent of other prognostic markers (Breslow thickness and ulceration) in this study. These findings applied equally to UV-exposed and non-UV-exposed body sites, highlighting the need for whole-body examinations in patients with a history of CSD. Our data suggest that the relative risk from sun exposure (intermittent and chronic) differs with respect to different subtypes of malignant melanomas.

## Figures and Tables

**Table 1:** Clinical characteristics of patients included in the histopathological analysis. Abbreviations: UV—ultraviolet; AE—actinic elastosis; TEG—tumor elastosis grade.

	Nevi (*n* = 92)	Melanomas (*n* = 503)	*p*
Subtype of melanoma	-	SSM = 167 (33.2%) NMM = 199 (39.6%) LMM = 91 (18.1%) ALM = 27 (5.4%) Other/unspecified = 19 (3.8%)	-
Sex	Male = 30 (32.6%) Female = 62 (67.4%)	Male = 296 (58.8%) Female = 207 (41.2%)	-
Age at diagnosis (y)	46.0 (±15.7)	68.9 (±14.5)	≤0.001
UV-exposed body site	16 (17.4%)	159 (31.6%)	≤0.001
Mean depth of AE (mm)	0.27 (±0.28)	0.62 (±0.43)	≤0.001
TEG (mm)	0.31 (±0.56)	1.05 (±1.16)	≤0.001

**Table 2:** Clinical characteristics of patients included in the histopathological analysis, with a focus on LMM compared to other subtypes of melanomas. Abbreviations: UV—ultraviolet; AE—actinic elastosis; TEG—tumor elastosis grade.

Category	Lentigo Maligna Melanoma (*n* = 91)	Other Subtypes of Melanomas (*n* = 412)	*p*
Sex	male = 47 (51.6%) female = 44 (48.4%)	male = 249 (60.4%) female = 163 (39.6%)	
Age at diagnosis (y)	76.1 (±9.8)	67.2 (±14.8)	≤0.001
UV-exposed body site	77 (84.6%)	82 (19.9%)	≤0.001
Head and neck	73 (80.2%)	62 (15%)	≤0.001
Mean depth of AE (mm)	0.96 (±0.38)	0.54 (±0.40)	≤0.001
TEG (mm)	2.22 (±1.26)	0.80 (±0.96)	≤0.001

**Table 3:** Multiple regression identified a correlation between depth of AE and TEG. Statistically significant results were found for the clinical subtype of melanoma (LMM vs. other subtypes), age at time of diagnosis, and UV-exposed body sites. Abbreviations: AE—actinic elastosis; TEG—tumor elastosis grade.

Depth of AE				TEG		
Category	Unstandardized Coefficients		Statistics	Unstandardized Coefficients		Statistics
	B	Std. Error	*p*	B	Std. Error	*p*
LMM vs. MM	0.123	0.047	0.008	0.521	0.117	<0.001
Age at diagnosis	0.012	0.001	<0.001	0.027	0.003	<0.001
UV-exposed body site	0.285	0.038	<0.001	1.031	0.096	<0.001

**Table 4:** Multiple regression showed a correlation between depth of AE and TEG. Statistically significant results were found for age at time of diagnosis and UV-exposed body sites. Only depth of AE showed significant results for clinical subtype of melanoma (SSM vs. NMM), but not for TEG.

Depth of AE				TEG		
Category	Unstandardized Coefficients		Statistics	Unstandardized Coefficients		Statistics
	B	Std. Error	*p*	B	Std. Error	*p*
SSM vs. NMM	0.089	0.033	0.007	0.093	0.080	0.246
Age at diagnosis	0.012	0.001	<0.001	0.026	0.003	<0.001
UV-exposed body site	0.278	0.042	<0.001	1.088	0.102	<0.001

## Data Availability

The data presented in this study are available from the corresponding author upon request.
